# Discovery of Single Nucleotide Polymorphisms and Mutations by Pyrosequencing

**DOI:** 10.1002/cfg.132

**Published:** 2002-02

**Authors:** Mostafa Ronaghi, Elahe Elahi

**Affiliations:** 1 Stanford Genome Technology Center 855 California Avenue Palo Alto CA 94304 USA; 2 Faculty of Science University of Tehran Tehran Iran; 3 Stanford Genome Technology Center Stanford University 855 California Avenue Palo Alto CA 94304 USA

## Abstract

Comparative genomics, analyzing variation among individual genomes, is an area of intense investigation. DNA sequencing is usually employed to look for polymorphisms and mutations. Pyrosequencing, a real-time DNA sequencing method, is emerging as a popular platform for comparative genomics. Here we review the use of this technology for mutation scanning, polymorphism discovery and chemical haplotyping. We describe the methodology and accuracy of this technique and discuss how to reduce the cost for large-scale analysis.


**Comparative and Functional Genomics**
*Comp Funct Genom* 2002; **3**: 51–56.
**DOI:** 10.1002/cfg.132

**Review**

### Discovery of single nucleotide polymorphisms and mutations by Pyrosequencing

Mostafa Ronaghi[1]* and Elahe Elahi[1,2]
[1] *Stanford Genome Technology Center 855 California Ave. Palo Alto, CA 94304, USA*
[2] *Faculty of Science, University of Tehran, Tehran, Iran*

**Correspondence to:*
*Stanford Genome Technology Center, Stanford University, 855 California Avenue, Palo Alto CA 94304, USA.*
*E-mail: mostafa@stanford.edu*

Received: 23 October 2001
Accepted: 27 November 2001
Published online:
11 January 2002

### Abstract

**Comparative genomics, analyzing variation among individual genomes, is an area of intense investigation. DNA sequencing is usually employed to look for polymorphisms and mutations. Pyrosequencing, a real-time DNA sequencing method, is emerging as a popular platform for comparative genomics. Here we review the use of this technology for mutation scanning, polymorphism discovery and chemical haplotyping. We describe the methodology and accuracy of this technique and discuss how to reduce the cost for large-scale analysis.**
**Copyright © 2002 John Wiley & Sons, Ltd.**

**Keywords:** SNP; Pyrosequencing; re-sequencing; bioluminescence; haplotyping; mutation detection; luciferase

Determination of mutations and polymorphisms in a genome is one of the most important tasks in the studies of biological systems today. Three DNA sequencing platforms are now being used to scan for mutations and polymorphisms. These include Sanger DNA sequencing [22] hybridization-based sequencing [4,6,10,23,24] and Pyrosequencing [17,20]. Pyrosequencing is based on the detection of released pyrophosphate (PPi) during DNA synthesis. In a cascade of enzymatic reactions, visible light proportional to the number of incorporated nucleotides is generated (Figure 1). The cascade starts with a nucleic acid polymerization reaction in which inorganic pyrophosphate (PPi) is released as a result of nucleotide incorporation by polymerase. The released PPi is subsequently converted to ATP by ATP sulfurylase which provides the energy to luciferase to oxidize luciferin and generate light. Since the added nucleotide is known, the sequence of the template can be determined (Figure 1). Pyrosequencing has the potential advantages of accuracy, flexibility, parallel processing and can be easily automated. Furthermore it dispenses with the need for labelled primers, labelled nucleotides and gel-electrophoresis [21]. The methodological performance of Pyrosequencng in determination of difficult secondary DNA structures [19], mutation detection [2], cDNA analysis [12,14,18], re-sequencing of disease-associated genes [3,8], bacterial typing [11], viral typing [9] and single-nucleotide polymorphism analysis [1,5,7] has been shown. Most recently, we reported on multiplexing of Pyrosequencing [15] and showed the usefulness of single-stranded DNA-binding protein in the Pyrosequencing reaction system for long read sequencing and sequence determination of difficult DNA templates [16].

Current Pyrosequencing strategy using a commercial machine allows more than 50 nucleotides to be *de novo* sequenced routinely. Pyrosequencing may be the method of choice for sequencing of difficult secondary DNA structures which cannot be sequenced by conventional sequencing. In this review we discuss the use of Pyrosequencing for mutation scanning, SNP scanning and haplotyping, and describe the cost reduction efforts for large-scale studies.

### Re-sequencing for mutation discovery

The ability to sequence a large number of DNA samples rapidly and accurately for detection of all possible mutations is a critical goal in biomedicine.

Copyright © 2002 John Wiley & Sons, Ltd.

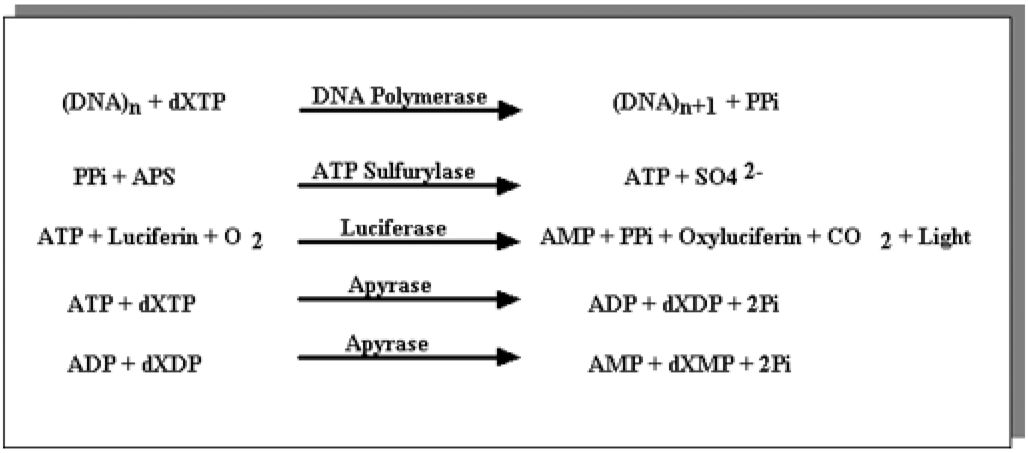

**Figure 1.** The general principle behind Pyrosequencing. A polymerase catalyzes incorporation of nucleotide(s) into a nucleic acid chain. As a result of the incorporation a PPi molecule(s) is released and subsequently converted to ATP by ATP sulfurylase. Light is produced in the luciferase reaction during which a luciferin molecule is oxidized. Apyrase degrades unused ATP and dNTP prior to the addition of the next nucleotide

DNA mutations can be classified as known or unknown mutations. If the mutation is known, the region containing the mutation can be analyzed at or nearby the mutation site. If the mutation is unknown, re-sequencing of the region for determination of the nature of the mutation is required. When DNA from biopsy material is being re-sequenced, a quantitative method for determining the ratio between wild and mutated template is desired. When analyzing heterozygous samples, conventional DNA sequencing does not reveal the exact ratio of mutated DNA to wild type and quite often cannot even detect the mutation when the ratio is below 0.5. However, Pyrosequencing has been shown to produce quantitative data and has been used to detect alleles with a frequency as low as 5% (www.pyrosequencing.com). Although this accuracy is only obtained with known polymorphisms, a ratio of 0.3 can be detected with a relatively high accuracy while scanning for mutation (Figure 2). We recently reported on the use of this technology for mutation scanning of the p53 gene in DNA extracted from biopsies and could detect new mutations in blind tests [8]. Pyrosequencing has also been used in mutation scanning of mitochondrial DNA (Figure 3). The sensitivity in mutation detection may be improved by the use of specially designed software programs for comparison of the obtained sequencing data with a reference data. When scanning for mutation in disease-associated DNA samples, a programmed dispensing order can be used allowing longer reads to be obtained which facilitates mutation detection. In addition, Pyrosequencing analysis has the potential to determine the allelic distribution of mutations in samples, which carry more than one mutation. This information could contribute to a better understanding of the effects of gene alterations in different diseases and lead to improved clinical interpretation.

### Single nucleotide polymorphism discovery

The Pyrosequencing strategy using commercial machines allows 60 nucleotides to be sequenced routinely. This will allow scanning for polymorphisms across a DNA template. Figure 4 demonstrates polymorphism scanning on a 500 nucleotide long DNA fragment. An average read-length of more than 60 nucleotides was obtained and we successfully detected the single nucleotide polymorphisms. Both homozygous (Figure 4a) and heterozygous (Figure 4b) templates were sequenced. Comparison of the sequences were performed manually, however, a higher accuracy in SNP discovery can be obtained when the pyrograms are compared by specialized software.

### Haplotyping

Pyrosequencing is based on sequencing-by-synthesis. Therefore, different phases at polymorphic regions

Copyright © 2002 John Wiley & Sons, Ltd.

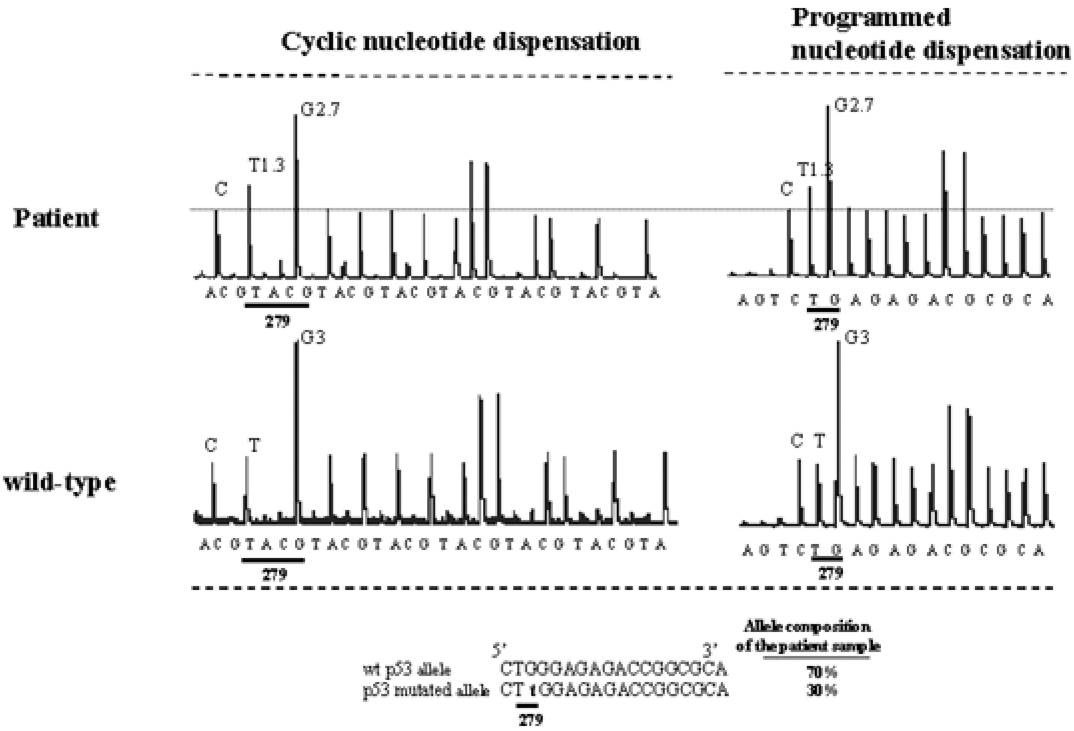

**Figure 2.** Pyrograms obtained from p53 mutation scanning. Comparison between Pyrosequencing results obtained by the cyclic and the programmed nucleotide dispensation approaches performed on exon 8 of the human p53 gene from wild-type and patient material. An AG to TG substitution in 30% of the alleles of the patient material was clearly detected by both approaches. Reprinted from *Gene*, 253(2), Garcia CA. *et al.* Mutation detection by pyrosequencing: sequencing of exons 5–8 of the p53 tumor suppressor gene, 247–257. Copyright © 2000, with permission from Elsevier Science

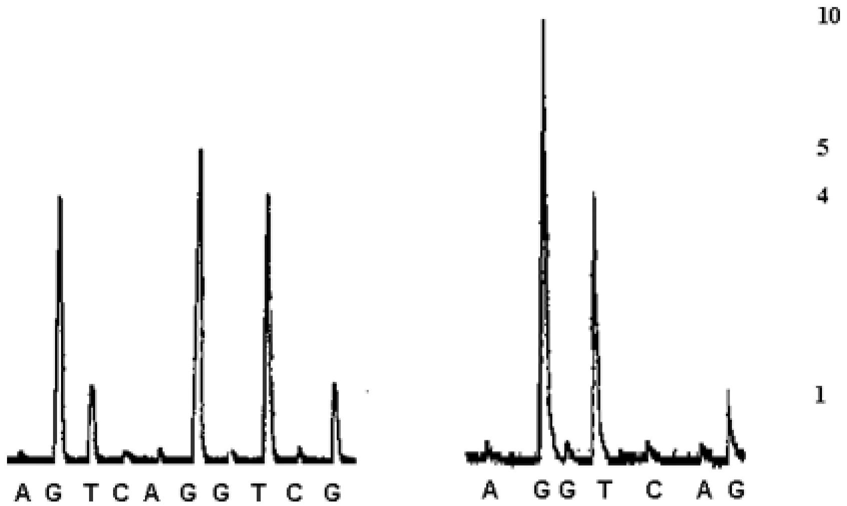

**Figure 3.** Pyrograms demonstrating raw data obtained on a 500 nucleotide-long PCR product obtained from D-loop of mitochondrial DNA from two different individuals. The correct sequence for the left pyrogram is GGGGTGGGGGTTTTG and the sequence for the right pyrogram is GGGGGGGGGGTTTTG

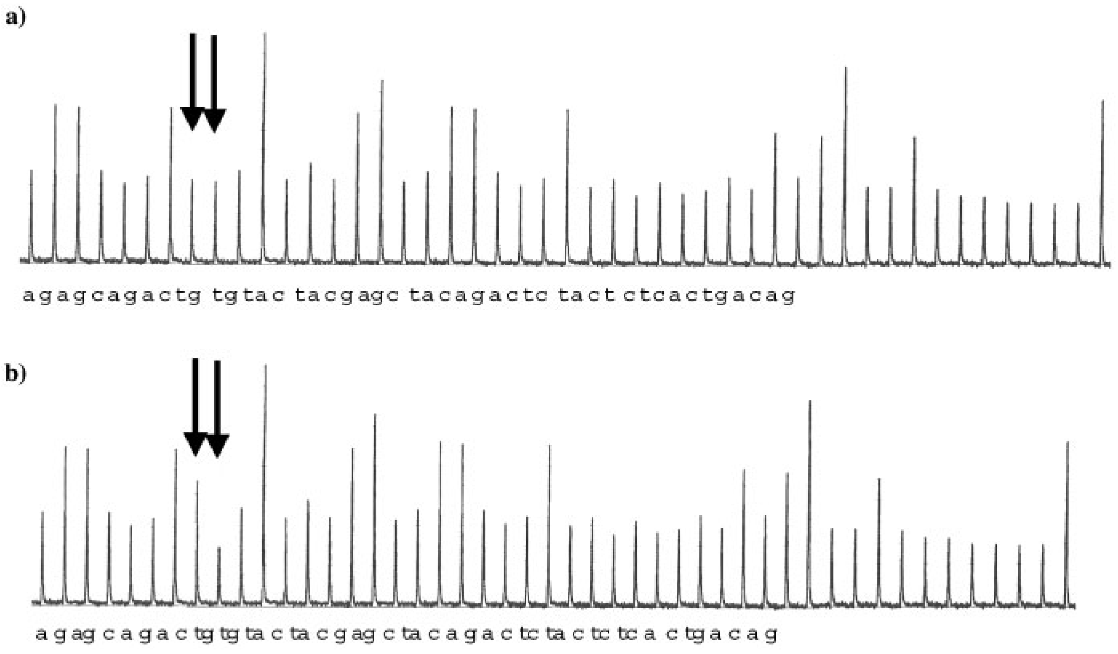

**Figure 4.** Pyrograms showing the discovery of a polymorphism on a 300 nucleotide-long DNA template using a programmed dispensation order. a) Homozygote b) Heterozygote. The arrows indicate the nucleotide position where the variation is seen. The nucleotide sequence is aggaagcaggaa/ctgggtgtaacctaccggagcttacagactcttactttctc-cactgacaggg where the polymorphic position is shown in bold may occur upon primer extension when sequencing heterozygous samples. This unique feature can be used for determination of SNP phases when the SNPs are in the vicinity of each other, thereby allowing the detection of haplotypes (Figure 5). Currently the read-length of Pyrosequencing is limited to 50–70 nucleotides and therefore it might be possible to determine the phase of SNPs if they are located within this range. Specialized software is under development to automatically determine the phase of SNPs.

### Challenges in reading polymorphic regions by Pyrosequencing

It is possible to detect polymorphisms when using Pyrosequencing as a platform for SNP discovery. If the polymorphism is a substitution, it will be possible to obtain a synchronized extension after the substituted nucleotide. If the polymorphism is a deletion or insertion of the same kind as the adjacent nucleotide on the DNA template, the sequence after the polymorphism will be synchronized. However, if the polymorphism is a deletion or insertion of another type the sequencing reaction can become out of phase making the interpretation of the subsequent sequence difficult. If the polymorphism is known, it is always possible to use programmed nucleotide delivery to keep the extension of different alleles synchronized after the polymorphic region. It is also possible to use a bi-directional approach [19] wherein the complementary strand is sequenced in order to decipher the sequence flanking the polymorphism.

Another inherent problem in Pyrosequencing is the difficulty in determining the number of incorporated nucleotides in homopolymeric regions due to the non-linear light response following incorporation of more than 5–6 identical nucleotides. The polymerization efficiency of eight sequential G nucleotides and ten sequential G nucleotides is demonstrated in Figures 4 and 3 respectively. However to elucidate the correct number of incorporated nucleotides it may be necessary to use specific software algorithms that integrate the signals. For re-sequencing it is possible to add the nucleotide twice for a homopolymeric region to ensure complete polymerization as demonstrated in Figures 4 and 3.

Copyright © 2002 John Wiley & Sons, Ltd. 

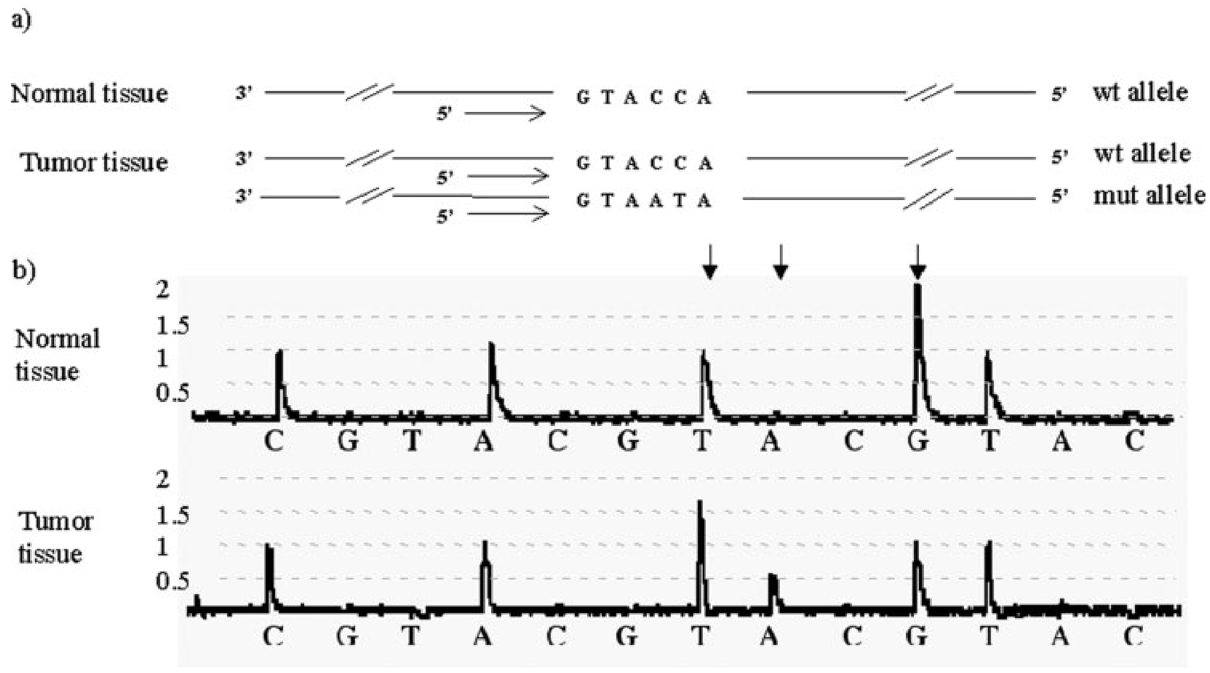

**Figure 5.** Traces demonstrating allelic determination from a polymorphic region in exon 5 of the p53 gene. a) The sequences of normal and tumor tissues. b) The occurrence of a 50% double mutation as 1.5 base signal for T with subsequent 0.5 base signal for A and 1 base signal for G is obtained. The result clearly demonstrates that both mutations reside on the same allele. Copyright © 2000 BioTechniques. Reproduced with permission

### Software for pyrogram analysis

SQA Software was recently developed to analyze tag sequences obtained by Pyrosequencing (**www.pyrosequencing.com**). The software operates under Windows® 2000 system and provides a base-calling algorithm which automatically scores the nucleotide sequence and calculates a quality value, which is displayed as a color code for each nucleotide scored. The assignment of quality values is based on a number of different parameters including difference in match between the best and next best choice of nucleotide peak, agreement between expected and obtained sequence around each peak, signal-to-noise ratios variance in peak heights in the sequence and peak width. The software also calculates out of phase signals to produce a synchronized processed sequence. In addition, the tag software allows multiple additions of the same nucleotide to ensure complete polymerization in homopolymeric regions. Currently, the software does not provide comparison of pyrograms which would be useful for polymorphism discovery and mutation scanning.

### Cost reduction efforts in Pyrosequencing technology

The cost for analysis of samples can be reduced by either improving the technology or decreasing the use of chemicals. We have recently developed and improved Pyrosequencing technology through reducing the cost per analysis. Most notably are developments of multiplex Pyrosequencing [15], use of a three primer system for amplification [7], development of enzymatic template preparation strategies [13,14] and use of Sepharose beads for immobilization of PCR products for Pyrosequencing (**www.pyrosequencing.com**). Another approach for cost reduction is to decrease the volume of the reaction and thereby to use less chemicals. Development of a 384-well based Pyrosequencing machine (PTP 384) has lowered the cost at least four folds. It is expected that miniaturization will reduce the cost for Pyrosequencing chemicals by one to three orders of magnitude. We are currently working on microfluidics and array formats for low volume Pyrosequencing analysis.

Copyright © 2002 John Wiley & Sons, Ltd. 

### Acknowledgement

The authors are supported by an NIH grant number 2P0I HG00205 and University of Tehran.

### References

1. Ahmadian A, Gharizadeh B, Gustafsson AC, *et al.* 2000a. Single-nucleotide polymorphism analysis by Pyrosequencing. *Anal Biochem* **280**: 103–110.
2. Ahmadian A, Lundeberg J, Nyren P, Uhlen M, Ronaghi M. 2000b. Analysis of the p53 tumor suppressor gene by pyrosequencing. *BioTechniques* **28**: 140–144.
3. Andreasson H, Asp A, Alderborn A, Gyllensten U, Allen M. 2002. A mitochondrial DNA typing method for forensic identification using Pyrosequencing technology. *Biotechniques* In press.
4. Bains W, Smith GC. 1988. A novel method for nucleic acid sequence determination. *J Theoret Biol* **135**: 303–307.
5. Eckersten A, Örlefors AE, Ellström C, *et al.* 2000. High-throughput SNP scoring in a disposable microfabricated CD device. *Proceeding of the Micro Total Analysis Systems*. Kluwer Academic Publishers: 521–524.
6. Drmanac R, Labat I, Brukner I, Crkvenjakov R. 1989. Sequencing of megabase plus DNA by hybridization: theory of the method. *Genomics* **4**: 114–128.
7. Fakhrai-Rad H, Pourmand N, Ronaghi M. 2002. Pyrosequencing: an accurate platform for analyzing of single nucleotide polymorphism. *Human Mutations* In Press.
8. Garcia AC, Ahmadian A, Gharizadeh B, Lundeberg J, Ronaghi M, Nyren P. 2000. Mutation detection by Pyrosequencing: sequencing of exons 5 to 8 of the p53 tumour supressor gene. *Gene* **253**: 249–257.
9. Gharizadeh B, Kalantari M, Garcia CA, Johansson B, Nyren P. 2001. Typing of human papillomavirus by pyrosequencing. *Lab Invest* **81**: 673–679.
10. Khrapko KR, Lysov YP, Khorlyn AA, Shick VV, Florentiev VL, Mirzabekov AD. 1989. An oligonucleotide hybridization approach to DNA sequencing. *FEBS Lett* **256**: 118–122.
11. Monstein H, Nikpour-Badr S, Jonasson J. 2001. Rapid molecular identification and subtyping of Helicobacter pylori by pyrosequencing of the 16S rDNA variable V1 and V3 regions. *FEMS Microbiol Lett* **15**: 103–107.
12. Nordstrom T, Nourizad K, Ronaghi M, Nyren P. 2000a. Methods enabling Pyrosequencing on double-stranded DNA. *Anal Biochem* **282**: 186–193.
13. Nordstrom T, Ronaghi M, Forsberg L, de Faire U, Morgenstern R, Nyren P. 2000b. Direct analysis of single-nucleotide polymorphism on double-stranded DNA by pyrosequencing. *Biotechnol Appl Biochem* **31**: 107–112.
14. Nordstrom T, Gharizadeh B, Pourmand N, Nyren P, Ronaghi M. 2001. Method enabling fast partial sequencing of cDNA clones. *Anal Biochem.* **292**: 266–271.
15. Pourmand N, Cheung R, Rofoogaran A, *et al.* 2001. Multiplex Pyrosequencing for viral typing. *Nucleic Acids Res In press.*
16. Ronaghi M. 2000. Improved performance of Pyrosequencing using single-stranded DNA-binding protein. *Anal Biochem* **286**: 282–288.
17. Ronaghi M, Karamohamed S, Pettersson B, Uhlen M, Nyren P. 1996. Real-time DNA sequencing using detection of pyrophosphate release. *Anal Biochem* **242**: 84–89.
18. Ronaghi M, Pettersson B, Uhlen M, Nyren P. 1998. PCR-introduced loop structure as primer in DNA sequencing. *BioTechniques* **25**: 876–884.
19. , Ronaghi M, Nygren M, Lundeberg J, Nyren P. 1999. Analyses of secondary structures in DNA by pyrosequencing. *Anal Biochem* **267**: 65–71.
20. Ronaghi M, Uhlen M, Nyren P. 1998. A sequencing method based on real-time pyrophosphate. *Science* **281**: 363–365.
21. Ronaghi M. 2001. Pyrosequencing sheds light on DNA sequencing. *Genome Res* **11**: 3–11.
22. Sanger F, Nicklen S, Coulson AR. 1977. DNA sequencing with chain-terminating inhibitors. *Proc Natl Acad Sci U S A* **74**: 5463–5467.
23. Southern EM. 1989. Analysing polynucleotide sequences. Patent WO/10977.
24. Strezoska Z, Paunesku T, Radosavljevic D, Labat I, Drmanac R, Crkvenjakov R. 1991. DNA sequencing by hybridization: 100 bases read by a non-gel-based method. *Proc Natl Acad Sci U S A* **88**: 10089–10093.

Copyright © 2002 John Wiley & Sons, Ltd. 
